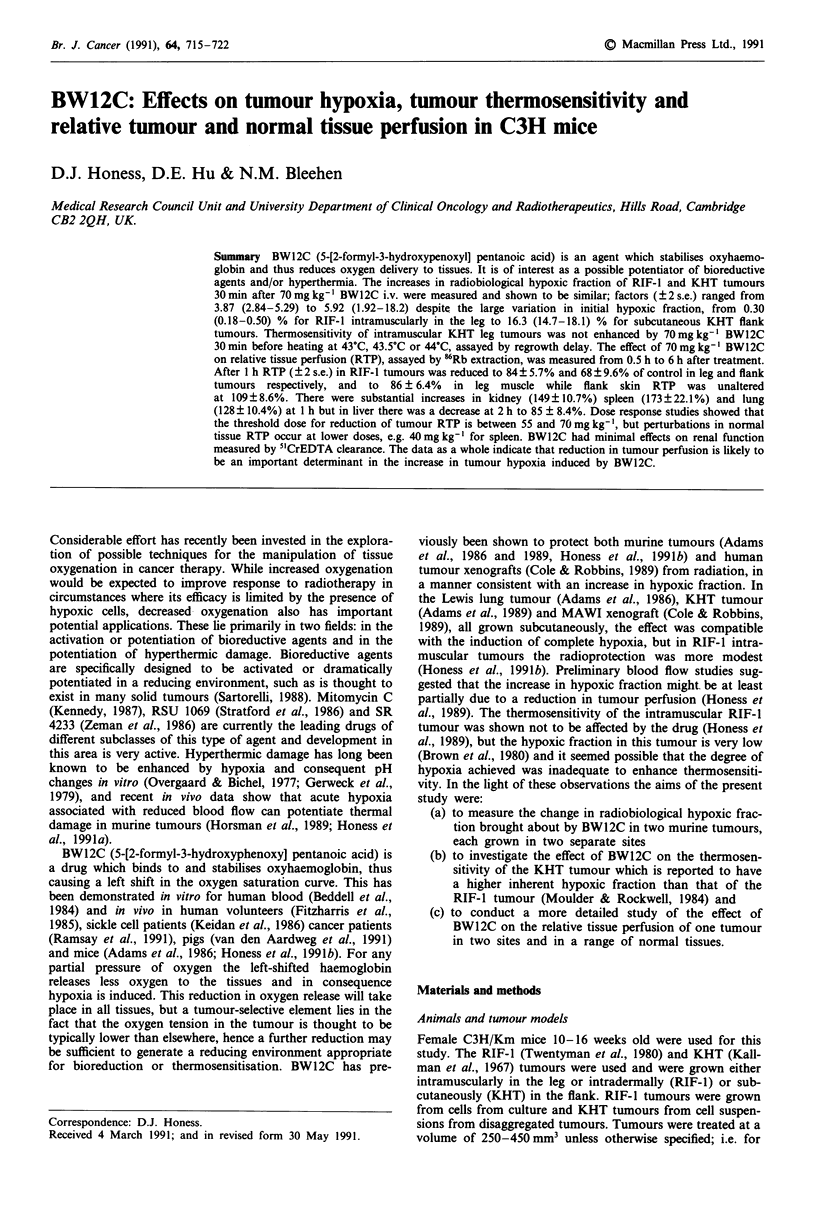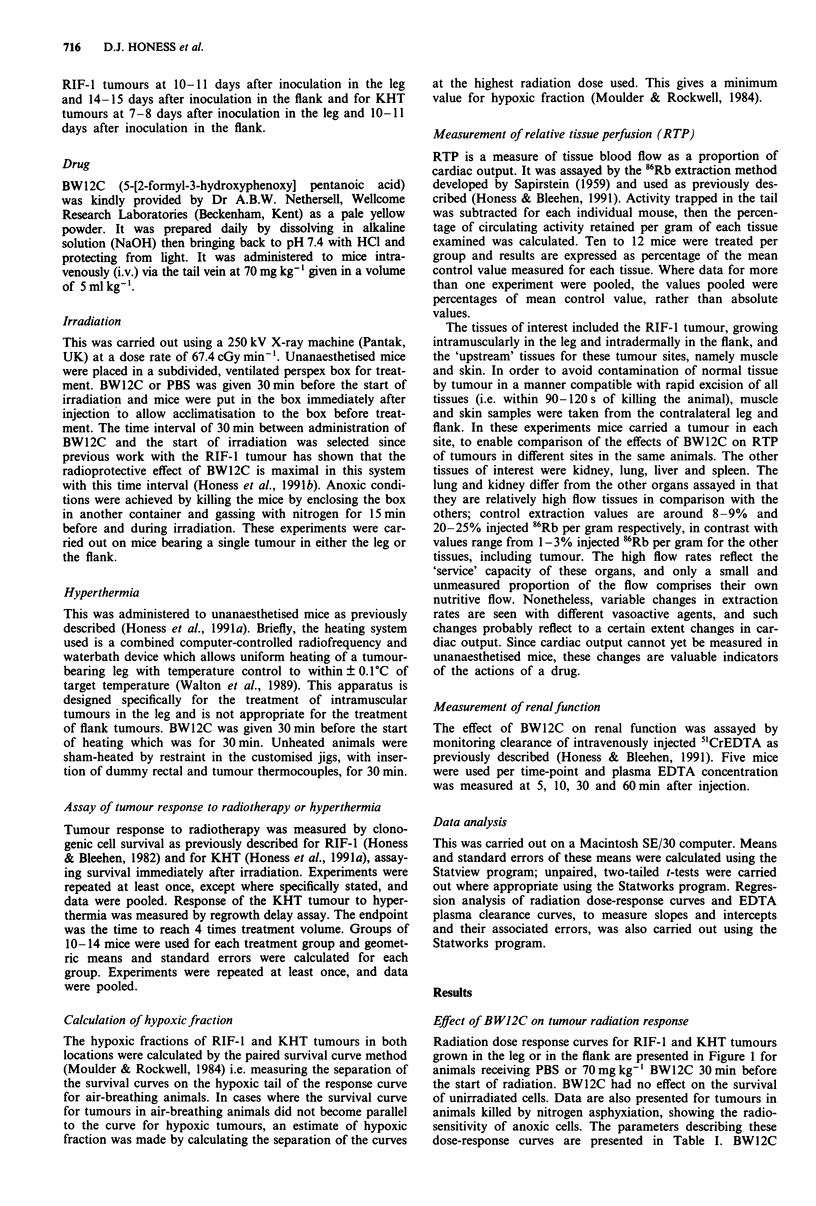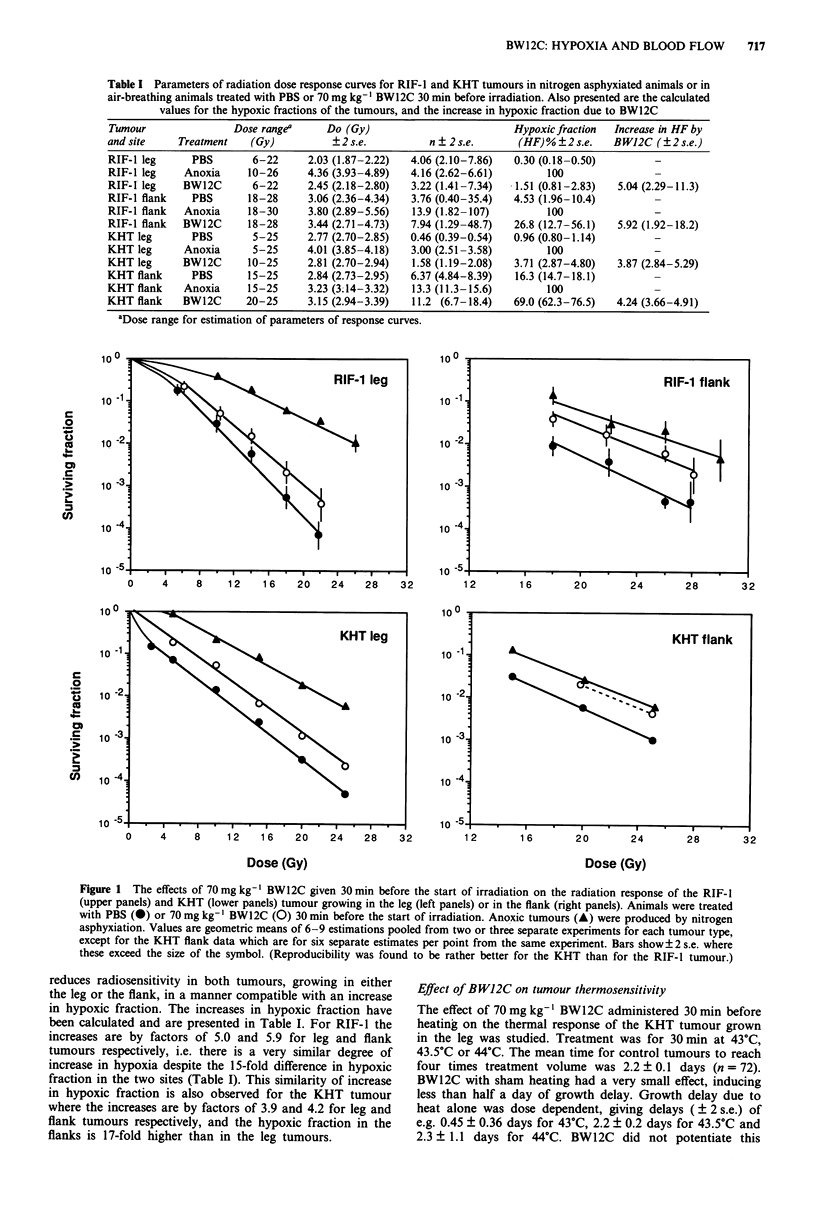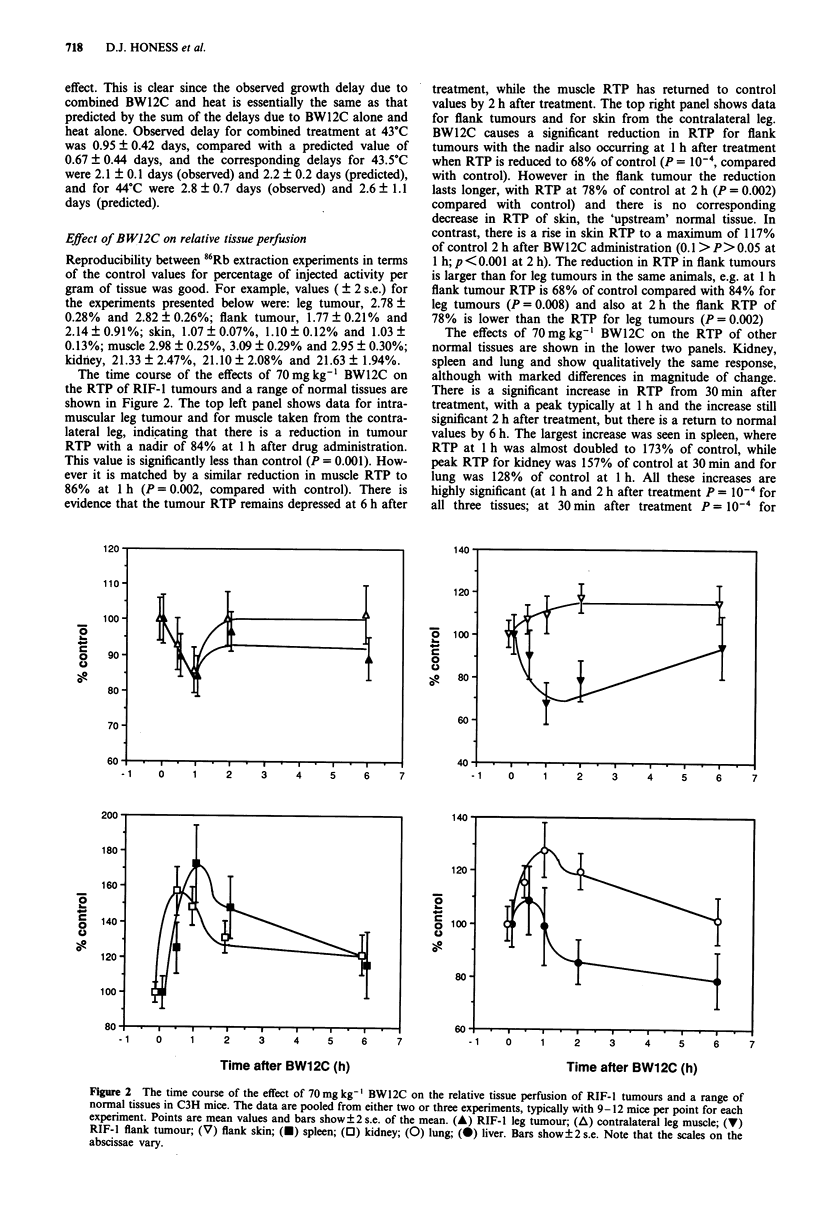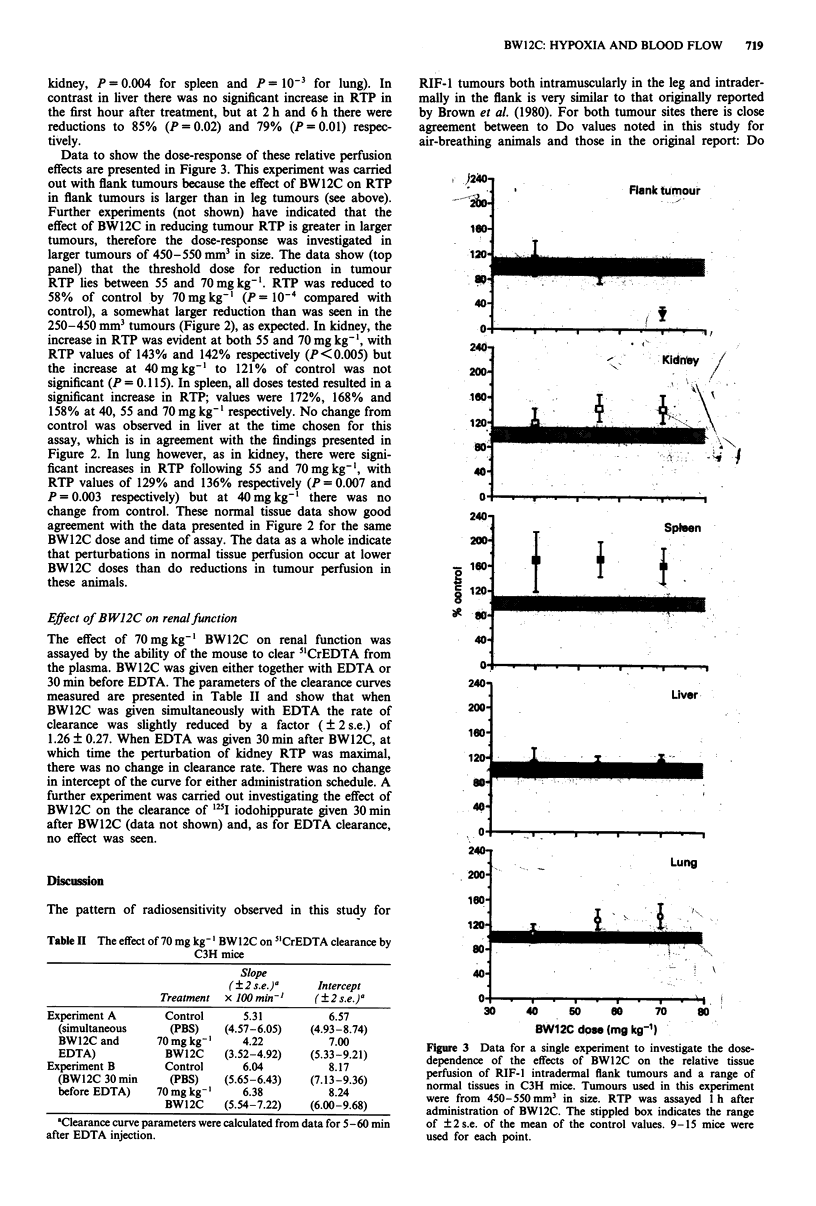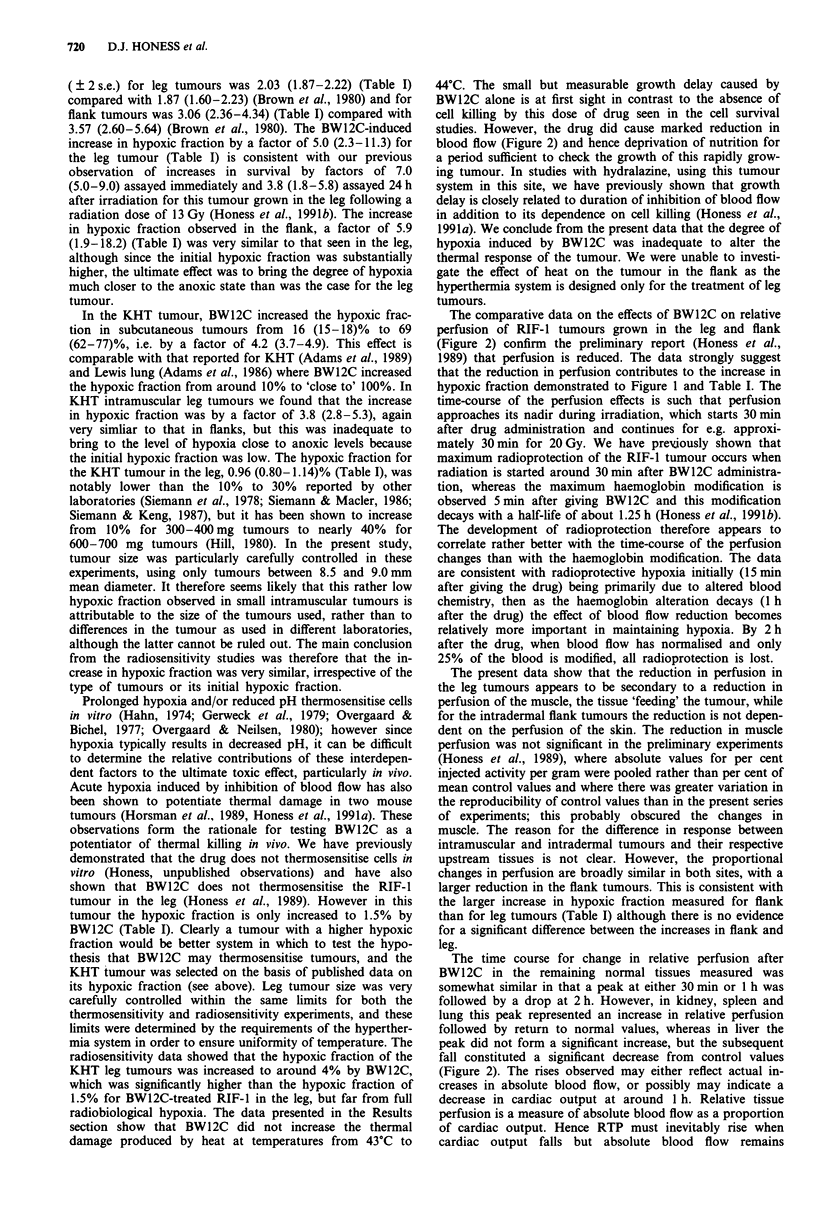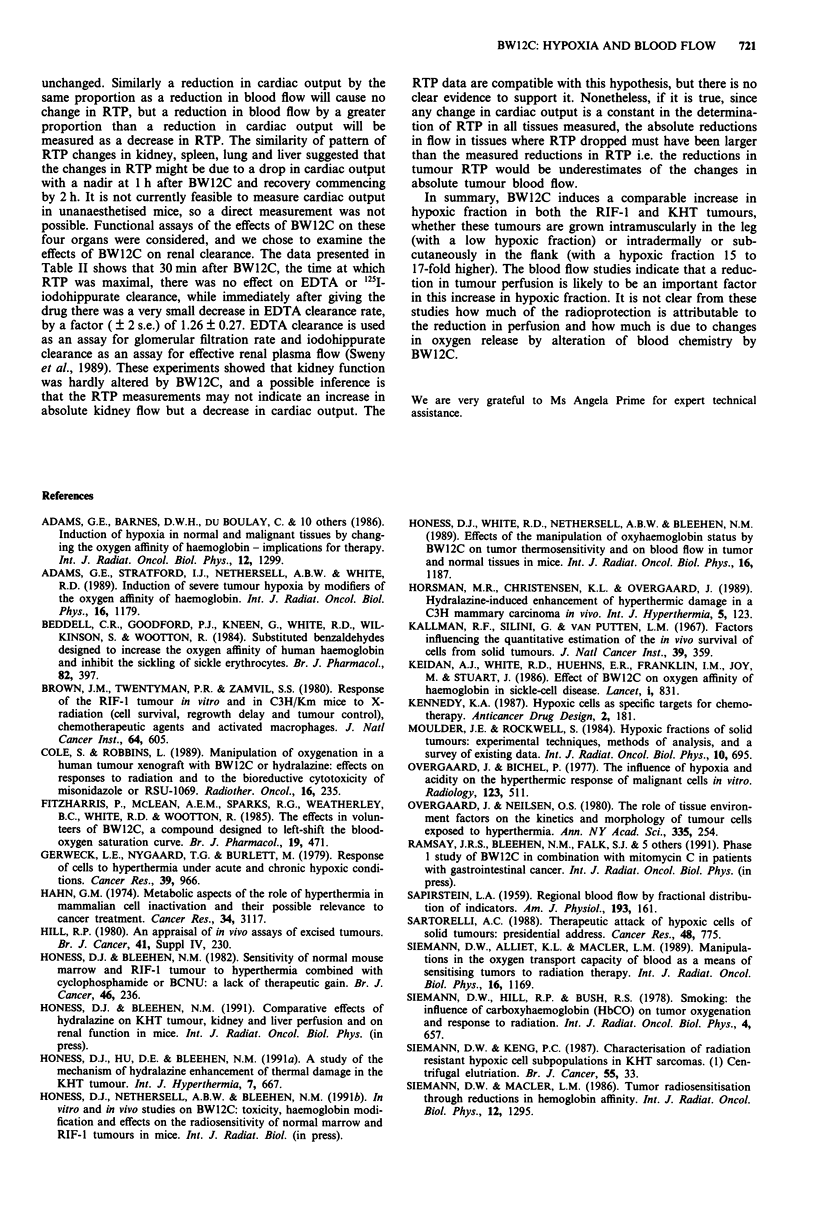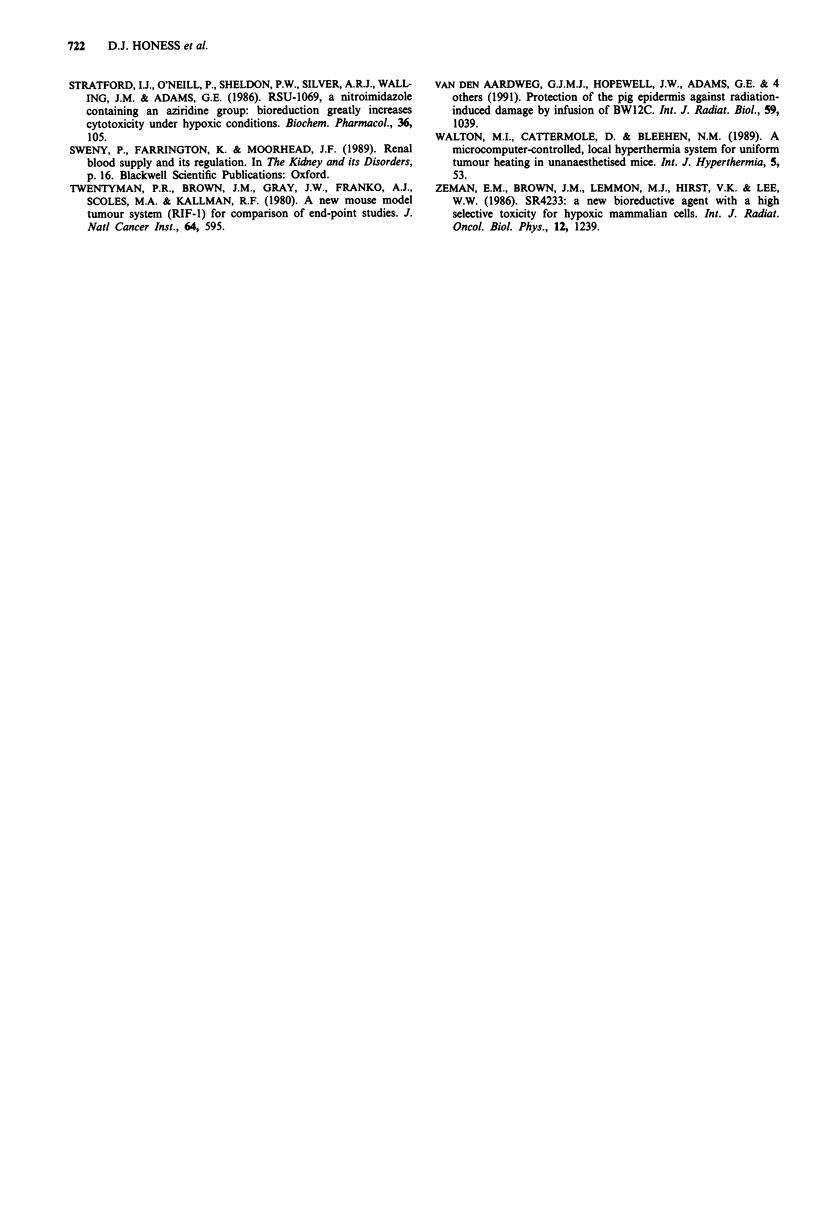# BW12C: effects on tumour hypoxia, tumour thermosensitivity and relative tumour and normal tissue perfusion in C3H mice.

**DOI:** 10.1038/bjc.1991.387

**Published:** 1991-10

**Authors:** D. J. Honess, D. E. Hu, N. M. Bleehen

**Affiliations:** Medical Research Council Unit, Cambridge, UK.

## Abstract

BW12C (5-[2-formyl-3-hydroxypenoxyl] pentanoic acid) is an agent which stabilises oxyhaemoglobin and thus reduces oxygen delivery to tissues. It is of interest as a possible potentiator of bioreductive agents and/or hyperthermia. The increases in radiobiological hypoxic fraction of RIF-1 and KHT tumours 30 min after 70 mg kg-1 BW12C i.v. were measured and shown to be similar; factors (+/- 2 s.e.) ranged from 3.87 (2.84-5.29) to 5.92 (1.92-18.2) despite the large variation in initial hypoxic fraction, from 0.30 (0.18-0.50) % for RIF-1 intramuscularly in the leg to 16.3 (14.7-18.1) % for subcutaneous KHT flank tumours. Thermosensitivity of intramuscular KHT leg tumours was not enhanced by 70 mg kg-1 BW12C 30 min before heating at 43 degrees C, 43.5 degrees C or 44 degrees C, assayed by regrowth delay. The effect of 70 mg kg-1 BW12C on relative tissue perfusion (RTP), assayed by 86Rb extraction, was measured from 0.5 h to 6 h after treatment. After 1 h RTP (+/- 2 s.e.) in RIF-1 tumours was reduced to 84 +/- 5.7% and 68 +/- 9.6% of control in leg and flank tumours respectively, and to 86 +/- 6.4% in leg muscle while flank skin RTP was unaltered at 109 +/- 8.6%. There were substantial increases in kidney (149 +/- 10.7%) spleen (173 +/- 22.1%) and lung (128 +/- 10.4%) at 1 h but in liver there was a decrease at 2 h to 85 +/- 8.4%. Dose response studies showed that the threshold dose for reduction of tumour RTP is between 55 and 70 mg kg-1, but perturbations in normal tissue RTP occur at lower doses, e.g. 40 mg kg-1 for spleen. BW12C had minimal effects on renal function measured by 51CrEDTA clearance. The data as a whole indicate that reduction in tumour perfusion is likely to be an important determinant in the increase in tumour hypoxia induced by BW12C.


*Br. J. Cancer* (1991), **64**, 715–722 © Macmillan Press Ltd., 1991

### BW12C: Effects on tumour hypoxia, tumour thermosensitivity and relative tumour and normal tissue perfusion in C3H mice

D.J. Honess, D.E. Hu & N.M. Bleehen

*Medical Research Council Unit and University Department of Clinical Oncology and Radiotherapeutics, Hills Road, Cambridge CB2 2QH, UK.*

**Summary** BW12C (5-[2-formyl-3-hydroxypenoxyl] pentanoic acid) is an agent which stabilises oxyhaemoglobin and thus reduces oxygen delivery to tissues. It is of interest as a possible potentiator of bioreductive agents and/or hyperthermia. The increases in radiobiological hypoxic fraction of RIF-1 and KHT tumours 30 min after 70 mg kg$^{-1}$ BW12C i.v. were measured and shown to be similar; factors (±2 s.e.) ranged from 3.87 (2.84–5.29) to 5.92 (1.92–18.2) despite the large variation in initial hypoxic fraction, from 0.30 (0.18–0.50) % for RIF-1 intramuscularly in the leg to 16.3 (14.7–18.1) % for subcutaneous KHT flank tumours. Thermosensitivity of intramuscular KHT leg tumours was not enhanced by 70 mg kg$^{-1}$ BW12C 30 min before heating at 43°C, 43.5°C or 44°C, assayed by regrowth delay. The effect of 70 mg kg$^{-1}$ BW12C on relative tissue perfusion (RTP), assayed by $^{86}$Rb extraction, was measured from 0.5 h to 6 h after treatment. After 1 h RTP (±2 s.e.) in RIF-1 tumours was reduced to 84±5.7% and 68±9.6% of control in leg and flank tumours respectively, and to 86 ± 6.4% in leg muscle while flank skin RTP was unaltered at 109±8.6%. There were substantial increases in kidney (149±10.7%) spleen (173±22.1%) and lung (128±10.4%) at 1 h but in liver there was a decrease at 2 h to 85 ± 8.4%. Dose response studies showed that the threshold dose for reduction of tumour RTP is between 55 and 70 mg kg$^{-1}$, but perturbations in normal tissue RTP occur at lower doses, e.g. 40 mg kg$^{-1}$ for spleen. BW12C had minimal effects on renal function measured by $^{51}$CrEDTA clearance. The data as a whole indicate that reduction in tumour perfusion is likely to be an important determinant in the increase in tumour hypoxia induced by BW12C.

Considerable effort has recently been invested in the exploration of possible techniques for the manipulation of tissue oxygenation in cancer therapy. While increased oxygenation would be expected to improve response to radiotherapy in circumstances where its efficacy is limited by the presence of hypoxic cells, decreased oxygenation also has important potential applications. These lie primarily in two fields: in the activation or potentiation of bioreductive agents and in the potentiation of hyperthermic damage. Bioreductive agents are specifically designed to be activated or dramatically potentiated in a reducing environment, such as is thought to exist in many solid tumours (Sartorelli, 1988). Mitomycin C (Kennedy, 1987), RSU 1069 (Stratford *et al.*, 1986) and SR 4233 (Zeman *et al.*, 1986) are currently the leading drugs of different subclasses of this type of agent and development in this area is very active. Hyperthermic damage has long been known to be enhanced by hypoxia and consequent pH changes *in vitro* (Overgaard & Bichel, 1977; Gerweck *et al.*, 1979), and recent *in vivo* data show that acute hypoxia associated with reduced blood flow can potentiate thermal damage in murine tumours (Horsman *et al.*, 1989; Honess *et al.*, 1991*a*).

BW12C (5-[2-formyl-3-hydroxyphenoxy] pentanoic acid) is a drug which binds to and stabilises oxyhaemoglobin, thus causing a left shift in the oxygen saturation curve. This has been demonstrated *in vitro* for human blood (Beddell *et al.*, 1984) and *in vivo* in human volunteers (Fitzharris *et al.*, 1985), sickle cell patients (Keidan *et al.*, 1986) cancer patients (Ramsay *et al.*, 1991), pigs (van den Aardweg *et al.*, 1991) and mice (Adams *et al.*, 1986; Honess *et al.*, 1991*b*). For any partial pressure of oxygen the left-shifted haemoglobin releases less oxygen to the tissues and in consequence hypoxia is induced. This reduction in oxygen release will take place in all tissues, but a tumour-selective element lies in the fact that the oxygen tension in the tumour is thought to be typically lower than elsewhere, hence a further reduction may be sufficient to generate a reducing environment appropriate for bioreduction or thermosensitisation. BW12C has previously been shown to protect both murine tumours (Adams *et al.*, 1986 and 1989, Honess *et al.*, 1991*b*) and human tumour xenografts (Cole & Robbins, 1989) from radiation, in a manner consistent with an increase in hypoxic fraction. In the Lewis lung tumour (Adams *et al.*, 1986), KHT tumour (Adams *et al.*, 1989) and MAWI xenograft (Cole & Robbins, 1989), all grown subcutaneously, the effect was compatible with the induction of complete hypoxia, but in RIF-1 intramuscular tumours the radioprotection was more modest (Honess *et al.*, 1991*b*). Preliminary blood flow studies suggested that the increase in hypoxic fraction might be at least partially due to a reduction in tumour perfusion (Honess *et al.*, 1989). The thermosensitivity of the intramuscular RIF-1 tumour was shown not to be affected by the drug (Honess *et al.*, 1989), but the hypoxic fraction in this tumour is very low (Brown *et al.*, 1980) and it seemed possible that the degree of hypoxia achieved was inadequate to enhance thermosensitivity. In the light of these observations the aims of the present study were:

(a) to measure the change in radiobiological hypoxic fraction brought about by BW12C in two murine tumours, each grown in two separate sites
(b) to investigate the effect of BW12C on the thermosensitivity of the KHT tumour which is reported to have a higher inherent hypoxic fraction than that of the RIF-1 tumour (Moulder & Rockwell, 1984) and
(c) to conduct a more detailed study of the effect of BW12C on the relative tissue perfusion of one tumour in two sites and in a range of normal tissues.

### Materials and methods

### Animals and tumour models

Female C3H/Km mice 10–16 weeks old were used for this study. The RIF-1 (Twentyman *et al.*, 1980) and KHT (Kallman *et al.*, 1967) tumours were used and were grown either intramuscularly in the leg or intradermally (RIF-1) or subcutaneously (KHT) in the flank. RIF-1 tumours were grown from cells from culture and KHT tumours from cell suspensions from disaggregated tumours. Tumours were treated at a volume of 250–450 mm$^3$ unless otherwise specified; i.e. for

Correspondence: D.J. Honess.
Received 4 March 1991; and in revised form 30 May 1991.

RIF-1 tumours at 10–11 days after inoculation in the leg and 14–15 days after inoculation in the flank and for KHT tumours at 7–8 days after inoculation in the leg and 10–11 days after inoculation in the flank.

*Drug*

BW12C (5-[2-formyl-3-hydroxyphenoxy] pentanoic acid) was kindly provided by Dr A.B.W. Nethersell, Wellcome Research Laboratories (Beckenham, Kent) as a pale yellow powder. It was prepared daily by dissolving in alkaline solution (NaOH) then bringing back to pH 7.4 with HCl and protecting from light. It was administered to mice intravenously (i.v.) via the tail vein at 70 mg kg$^{-1}$ given in a volume of 5 ml kg$^{-1}$.

*Irradiation*

This was carried out using a 250 kV X-ray machine (Pantak, UK) at a dose rate of 67.4 cGy min$^{-1}$. Unanaesthetised mice were placed in a subdivided, ventilated perspex box for treatment. BW12C or PBS was given 30 min before the start of irradiation and mice were put in the box immediately after injection to allow acclimatisation to the box before treatment. The time interval of 30 min between administration of BW12C and the start of irradiation was selected since previous work with the RIF-1 tumour has shown that the radioprotective effect of BW12C is maximal in this system with this time interval (Honess *et al.*, 1991*b*). Anoxic conditions were achieved by killing the mice by enclosing the box in another container and gassing with nitrogen for 15 min before and during irradiation. These experiments were carried out on mice bearing a single tumour in either the leg or the flank.

*Hyperthermia*

This was administered to unanaesthetised mice as previously described (Honess *et al.*, 1991*a*). Briefly, the heating system used is a combined computer-controlled radiofrequency and waterbath device which allows uniform heating of a tumour-bearing leg with temperature control to within ± 0.1°C of target temperature (Walton *et al.*, 1989). This apparatus is designed specifically for the treatment of intramuscular tumours in the leg and is not appropriate for the treatment of flank tumours. BW12C was given 30 min before the start of heating which was for 30 min. Unheated animals were sham-heated by restraint in the customised jigs, with insertion of dummy rectal and tumour thermocouples, for 30 min.

*Assay of tumour response to radiotherapy or hyperthermia*

Tumour response to radiotherapy was measured by clonogenic cell survival as previously described for RIF-1 (Honess & Bleehen, 1982) and for KHT (Honess *et al.*, 1991*a*), assaying survival immediately after irradiation. Experiments were repeated at least once, except where specifically stated, and data were pooled. Response of the KHT tumour to hyperthermia was measured by regrowth delay assay. The endpoint was the time to reach 4 times treatment volume. Groups of 10–14 mice were used for each treatment group and geometric means and standard errors were calculated for each group. Experiments were repeated at least once, and data were pooled.

*Calculation of hypoxic fraction*

The hypoxic fractions of RIF-1 and KHT tumours in both locations were calculated by the paired survival curve method (Moulder & Rockwell, 1984) i.e. measuring the separation of the survival curves on the hypoxic tail of the response curve for air-breathing animals. In cases where the survival curve for tumours in air-breathing animals did not become parallel to the curve for hypoxic tumours, an estimate of hypoxic fraction was made by calculating the separation of the curves at the highest radiation dose used. This gives a minimum value for hypoxic fraction (Moulder & Rockwell, 1984).

*Measurement of relative tissue perfusion (RTP)*

RTP is a measure of tissue blood flow as a proportion of cardiac output. It was assayed by the $^{86}$Rb extraction method developed by Sapirstein (1959) and used as previously described (Honess & Bleehen, 1991). Activity trapped in the tail was subtracted for each individual mouse, then the percentage of circulating activity retained per gram of each tissue examined was calculated. Ten to 12 mice were treated per group and results are expressed as percentage of the mean control value measured for each tissue. Where data for more than one experiment were pooled, the values pooled were percentages of mean control value, rather than absolute values.

The tissues of interest included the RIF-1 tumour, growing intramuscularly in the leg and intradermally in the flank, and the 'upstream' tissues for these tumour sites, namely muscle and skin. In order to avoid contamination of normal tissue by tumour in a manner compatible with rapid excision of all tissues (i.e. within 90–120 s of killing the animal), muscle and skin samples were taken from the contralateral leg and flank. In these experiments mice carried a tumour in each site, to enable comparison of the effects of BW12C on RTP of tumours in different sites in the same animals. The other tissues of interest were kidney, lung, liver and spleen. The lung and kidney differ from the other organs assayed in that they are relatively high flow tissues in comparison with the others; control extraction values are around 8–9% and 20–25% injected $^{86}$Rb per gram respectively, in contrast with values range from 1–3% injected $^{86}$Rb per gram for the other tissues, including tumour. The high flow rates reflect the 'service' capacity of these organs, and only a small and unmeasured proportion of the flow comprises their own nutritive flow. Nonetheless, variable changes in extraction rates are seen with different vasoactive agents, and such changes probably reflect to a certain extent changes in cardiac output. Since cardiac output cannot yet be measured in unanaesthetised mice, these changes are valuable indicators of the actions of a drug.

*Measurement of renal function*

The effect of BW12C on renal function was assayed by monitoring clearance of intravenously injected $^{51}$CrEDTA as previously described (Honess & Bleehen, 1991). Five mice were used per time-point and plasma EDTA concentration was measured at 5, 10, 30 and 60 min after injection.

*Data analysis*

This was carried out on a Macintosh SE/30 computer. Means and standard errors of these means were calculated using the Statview program; unpaired, two-tailed *t*-tests were carried out where appropriate using the Statworks program. Regression analysis of radiation dose-response curves and EDTA plasma clearance curves, to measure slopes and intercepts and their associated errors, was also carried out using the Statworks program.

### Results

*Effect of BW12C on tumour radiation response*

Radiation dose response curves for RIF-1 and KHT tumours grown in the leg or in the flank are presented in Figure 1 for animals receiving PBS or 70 mg kg$^{-1}$ BW12C 30 min before the start of radiation. BW12C had no effect on the survival of unirradiated cells. Data are also presented for tumours in animals killed by nitrogen asphyxiation, showing the radiosensitivity of anoxic cells. The parameters describing these dose-response curves are presented in Table I. BW12C

**Table I** Parameters of radiation dose response curves for RIF-1 and KHT tumours in nitrogen asphyxiated animals or in air-breathing animals treated with PBS or 70 mg $kg^{-1}$ BW12C 30 min before irradiation. Also presented are the calculated values for the hypoxic fractions of the tumours, and the increase in hypoxic fraction due to BW12C

| *Tumour and site* | *Treatment* | *Dose range[a] (Gy)* | *Do (Gy) ±2 s.e.* | *n ± 2 s.e.* | *Hypoxic fraction (HF)% ± 2 s.e.* | *Increase in HF by BW12C (±2 s.e.)* |
|---|---|---|---|---|---|---|
| RIF-1 leg | PBS | 6–22 | 2.03 (1.87–2.22) | 4.06 (2.10–7.86) | 0.30 (0.18–0.50) | – |
| RIF-1 leg | Anoxia | 10–26 | 4.36 (3.93–4.89) | 4.16 (2.62–6.61) | 100 | – |
| RIF-1 leg | BW12C | 6–22 | 2.45 (2.18–2.80) | 3.22 (1.41–7.34) | 1.51 (0.81–2.83) | 5.04 (2.29–11.3) |
| RIF-1 flank | PBS | 18–28 | 3.06 (2.36–4.34) | 3.76 (0.40–35.4) | 4.53 (1.96–10.4) | – |
| RIF-1 flank | Anoxia | 18–30 | 3.80 (2.89–5.56) | 13.9 (1.82–107) | 100 | – |
| RIF-1 flank | BW12C | 18–28 | 3.44 (2.71–4.73) | 7.94 (1.29–48.7) | 26.8 (12.7–56.1) | 5.92 (1.92–18.2) |
| KHT leg | PBS | 5–25 | 2.77 (2.70–2.85) | 0.46 (0.39–0.54) | 0.96 (0.80–1.14) | – |
| KHT leg | Anoxia | 5–25 | 4.01 (3.85–4.18) | 3.00 (2.51–3.58) | 100 | – |
| KHT leg | BW12C | 10–25 | 2.81 (2.70–2.94) | 1.58 (1.19–2.08) | 3.71 (2.87–4.80) | 3.87 (2.84–5.29) |
| KHT flank | PBS | 15–25 | 2.84 (2.73–2.95) | 6.37 (4.84–8.39) | 16.3 (14.7–18.1) | – |
| KHT flank | Anoxia | 15–25 | 3.23 (3.14–3.32) | 13.3 (11.3–15.6) | 100 | – |
| KHT flank | BW12C | 20–25 | 3.15 (2.94–3.39) | 11.2 (6.7–18.4) | 69.0 (62.3–76.5) | 4.24 (3.66–4.91) |

[a]Dose range for estimation of parameters of response curves.

**Figure 1** The effects of 70 mg $kg^{-1}$ BW12C given 30 min before the start of irradiation on the radiation response of the RIF-1 (upper panels) and KHT (lower panels) tumour growing in the leg (left panels) or in the flank (right panels). Animals were treated with PBS (●) or 70 mg $kg^{-1}$ BW12C (○) 30 min before the start of irradiation. Anoxic tumours (▲) were produced by nitrogen asphyxiation. Values are geometric means of 6–9 estimations pooled from two or three separate experiments for each tumour type, except for the KHT flank data which are for six separate estimates per point from the same experiment. Bars show ± 2 s.e. where these exceed the size of the symbol. (Reproducibility was found to be rather better for the KHT than for the RIF-1 tumour.)

reduces radiosensitivity in both tumours, growing in either the leg or the flank, in a manner compatible with an increase in hypoxic fraction. The increases in hypoxic fraction have been calculated and are presented in Table I. For RIF-1 the increases are by factors of 5.0 and 5.9 for leg and flank tumours respectively, i.e. there is a very similar degree of increase in hypoxia despite the 15-fold difference in hypoxic fraction in the two sites (Table I). This similarity of increase in hypoxic fraction is also observed for the KHT tumour where the increases are by factors of 3.9 and 4.2 for leg and flank tumours respectively, and the hypoxic fraction in the flanks is 17-fold higher than in the leg tumours.

*Effect of BW12C on tumour thermosensitivity*

The effect of 70 mg $kg^{-1}$ BW12C administered 30 min before heating on the thermal response of the KHT tumour grown in the leg was studied. Treatment was for 30 min at 43°C, 43.5°C or 44°C. The mean time for control tumours to reach four times treatment volume was 2.2 ± 0.1 days ($n = 72$). BW12C with sham heating had a very small effect, inducing less than half a day of growth delay. Growth delay due to heat alone was dose dependent, giving delays (± 2 s.e.) of e.g. 0.45 ± 0.36 days for 43°C, 2.2 ± 0.2 days for 43.5°C and 2.3 ± 1.1 days for 44°C. BW12C did not potentiate this effect. This is clear since the observed growth delay due to combined BW12C and heat is essentially the same as that predicted by the sum of the delays due to BW12C alone and heat alone. Observed delay for combined treatment at 43°C was 0.95 ± 0.42 days, compared with a predicted value of 0.67 ± 0.44 days, and the corresponding delays for 43.5°C were 2.1 ± 0.1 days (observed) and 2.2 ± 0.2 days (predicted), and for 44°C were 2.8 ± 0.7 days (observed) and 2.6 ± 1.1 days (predicted).

*Effect of BW12C on relative tissue perfusion*

Reproducibility between $^{86}$Rb extraction experiments in terms of the control values for percentage of injected activity per gram of tissue was good. For example, values ( ± 2 s.e.) for the experiments presented below were: leg tumour, 2.78 ± 0.28% and 2.82 ± 0.26%; flank tumour, 1.77 ± 0.21% and 2.14 ± 0.91%; skin, 1.07 ± 0.07%, 1.10 ± 0.12% and 1.03 ± 0.13%; muscle 2.98 ± 0.25%, 3.09 ± 0.29% and 2.95 ± 0.30%; kidney, 21.33 ± 2.47%, 21.10 ± 2.08% and 21.63 ± 1.94%.

The time course of the effects of 70 mg kg$^{-1}$ BW12C on the RTP of RIF-1 tumours and a range of normal tissues are shown in Figure 2. The top left panel shows data for intramuscular leg tumour and for muscle taken from the contralateral leg, indicating that there is a reduction in tumour RTP with a nadir of 84% at 1 h after drug administration. This value is significantly less than control ($P = 0.001$). However it is matched by a similar reduction in muscle RTP to 86% at 1 h ($P = 0.002$, compared with control). There is evidence that the tumour RTP remains depressed at 6 h after treatment, while the muscle RTP has returned to control values by 2 h after treatment. The top right panel shows data for flank tumours and for skin from the contralateral leg. BW12C causes a significant reduction in RTP for flank tumours with the nadir also occurring at 1 h after treatment when RTP is reduced to 68% of control ($P = 10^{-4}$, compared with control). However in the flank tumour the reduction lasts longer, with RTP at 78% of control at 2 h ($P = 0.002$) compared with control) and there is no corresponding decrease in RTP of skin, the 'upstream' normal tissue. In contrast, there is a rise in skin RTP to a maximum of 117% of control 2 h after BW12C administration ($0.1 > P > 0.05$ at 1 h; $p < 0.001$ at 2 h). The reduction in RTP in flank tumours is larger than for leg tumours in the same animals, e.g. at 1 h flank tumour RTP is 68% of control compared with 84% for leg tumours ($P = 0.008$) and also at 2 h the flank RTP of 78% is lower than the RTP for leg tumours ($P = 0.002$)

The effects of 70 mg kg$^{-1}$ BW12C on the RTP of other normal tissues are shown in the lower two panels. Kidney, spleen and lung and show qualitatively the same response, although with marked differences in magnitude of change. There is a significant increase in RTP from 30 min after treatment, with a peak typically at 1 h and the increase still significant 2 h after treatment, but there is a return to normal values by 6 h. The largest increase was seen in spleen, where RTP at 1 h was almost doubled to 173% of control, while peak RTP for kidney was 157% of control at 30 min and for lung was 128% of control at 1 h. All these increases are highly significant (at 1 h and 2 h after treatment $P = 10^{-4}$ for all three tissues; at 30 min after treatment $P = 10^{-4}$ for

**Figure 2** The time course of the effect of 70 mg kg$^{-1}$ BW12C on the relative tissue perfusion of RIF-1 tumours and a range of normal tissues in C3H mice. The data are pooled from either two or three experiments, typically with 9–12 mice per point for each experiment. Points are mean values and bars show ± 2 s.e. of the mean. (▲) RIF-1 leg tumour; (△) contralateral leg muscle; (▼) RIF-1 flank tumour; (▽) flank skin; (■) spleen; (□) kidney; (○) lung; (●) liver. Bars show ± 2 s.e. Note that the scales on the abscissae vary.

kidney, $P = 0.004$ for spleen and $P = 10^{-3}$ for lung). In contrast in liver there was no significant increase in RTP in the first hour after treatment, but at 2 h and 6 h there were reductions to 85% ($P = 0.02$) and 79% ($P = 0.01$) respectively.

Data to show the dose-response of these relative perfusion effects are presented in Figure 3. This experiment was carried out with flank tumours because the effect of BW12C on RTP in flank tumours is larger than in leg tumours (see above). Further experiments (not shown) have indicated that the effect of BW12C in reducing tumour RTP is greater in larger tumours, therefore the dose-response was investigated in larger tumours of 450–550 $mm^3$ in size. The data show (top panel) that the threshold dose for reduction in tumour RTP lies between 55 and 70 mg $kg^{-1}$. RTP was reduced to 58% of control by 70 mg $kg^{-1}$ ($P = 10^{-4}$ compared with control), a somewhat larger reduction than was seen in the 250–450 $mm^3$ tumours (Figure 2), as expected. In kidney, the increase in RTP was evident at both 55 and 70 mg $kg^{-1}$, with RTP values of 143% and 142% respectively ($P < 0.005$) but the increase at 40 mg $kg^{-1}$ to 121% of control was not significant ($P = 0.115$). In spleen, all doses tested resulted in a significant increase in RTP; values were 172%, 168% and 158% at 40, 55 and 70 mg $kg^{-1}$ respectively. No change from control was observed in liver at the time chosen for this assay, which is in agreement with the findings presented in Figure 2. In lung however, as in kidney, there were significant increases in RTP following 55 and 70 mg $kg^{-1}$, with RTP values of 129% and 136% respectively ($P = 0.007$ and $P = 0.003$ respectively) but at 40 mg $kg^{-1}$ there was no change from control. These normal tissue data show good agreement with the data presented in Figure 2 for the same BW12C dose and time of assay. The data as a whole indicate that perturbations in normal tissue perfusion occur at lower BW12C doses than do reductions in tumour perfusion in these animals.

*Effect of BW12C on renal function*

The effect of 70 mg $kg^{-1}$ BW12C on renal function was assayed by the ability of the mouse to clear $^{51}$CrEDTA from the plasma. BW12C was given either together with EDTA or 30 min before EDTA. The parameters of the clearance curves measured are presented in Table II and show that when BW12C was given simultaneously with EDTA the rate of clearance was slightly reduced by a factor ($\pm 2$ s.e.) of $1.26 \pm 0.27$. When EDTA was given 30 min after BW12C, at which time the perturbation of kidney RTP was maximal, there was no change in clearance rate. There was no change in intercept of the curve for either administration schedule. A further experiment was carried out investigating the effect of BW12C on the clearance of $^{125}$I iodohippurate given 30 min after BW12C (data not shown) and, as for EDTA clearance, no effect was seen.

### Discussion

The pattern of radiosensitivity observed in this study for RIF-1 tumours both intramuscularly in the leg and intradermally in the flank is very similar to that originally reported by Brown *et al.* (1980). For both tumour sites there is close agreement between to Do values noted in this study for air-breathing animals and those in the original report: Do

**Table II** The effect of 70 mg $kg^{-1}$ BW12C on $^{51}$CrEDTA clearance by C3H mice

| | Treatment | *Slope* ($\pm 2$ *s.e.*)[a] × *100 min*$^{-1}$ | *Intercept* ($\pm 2$ *s.e.*)[a] |
|---|---|---|---|
| Experiment A | Control | 5.31 | 6.57 |
| (simultaneous | (PBS) | (4.57–6.05) | (4.93–8.74) |
| BW12C and | 70 mg $kg^{-1}$ | 4.22 | 7.00 |
| EDTA) | BW12C | (3.52–4.92) | (5.33–9.21) |
| Experiment B | Control | 6.04 | 8.17 |
| (BW12C 30 min | (PBS) | (5.65–6.43) | (7.13–9.36) |
| before EDTA) | 70 mg $kg^{-1}$ | 6.38 | 8.24 |
| | BW12C | (5.54–7.22) | (6.00–9.68) |

[a]Clearance curve parameters were calculated from data for 5–60 min after EDTA injection.

**Figure 3** Data for a single experiment to investigate the dose-dependence of the effects of BW12C on the relative tissue perfusion of RIF-1 intradermal flank tumours and a range of normal tissues in C3H mice. Tumours used in this experiment were from 450–550 $mm^3$ in size. RTP was assayed 1 h after administration of BW12C. The stippled box indicates the range of $\pm 2$ s.e. of the mean of the control values. 9–15 mice were used for each point.

(± 2 s.e.) for leg tumours was 2.03 (1.87–2.22) (Table I) compared with 1.87 (1.60–2.23) (Brown et al., 1980) and for flank tumours was 3.06 (2.36–4.34) (Table I) compared with 3.57 (2.60–5.64) (Brown et al., 1980). The BW12C-induced increase in hypoxic fraction by a factor of 5.0 (2.3–11.3) for the leg tumour (Table I) is consistent with our previous observation of increases in survival by factors of 7.0 (5.0–9.0) assayed immediately and 3.8 (1.8–5.8) assayed 24 h after irradiation for this tumour grown in the leg following a radiation dose of 13 Gy (Honess et al., 1991*b*). The increase in hypoxic fraction observed in the flank, a factor of 5.9 (1.9–18.2) (Table I) was very similar to that seen in the leg, although since the initial hypoxic fraction was substantially higher, the ultimate effect was to bring the degree of hypoxia much closer to the anoxic state than was the case for the leg tumour.

In the KHT tumour, BW12C increased the hypoxic fraction in subcutaneous tumours from 16 (15–18)% to 69 (62–77)%, i.e. by a factor of 4.2 (3.7–4.9). This effect is comparable with that reported for KHT (Adams et al., 1989) and Lewis lung (Adams et al., 1986) where BW12C increased the hypoxic fraction from around 10% to 'close to' 100%. In KHT intramuscular leg tumours we found that the increase in hypoxic fraction was by a factor of 3.8 (2.8–5.3), again very simliar to that in flanks, but this was inadequate to bring to the level of hypoxia close to anoxic levels because the initial hypoxic fraction was low. The hypoxic fraction for the KHT tumour in the leg, 0.96 (0.80–1.14)% (Table I), was notably lower than the 10% to 30% reported by other laboratories (Siemann et al., 1978; Siemann & Macler, 1986; Siemann & Keng, 1987), but it has been shown to increase from 10% for 300–400 mg tumours to nearly 40% for 600–700 mg tumours (Hill, 1980). In the present study, tumour size was particularly carefully controlled in these experiments, using only tumours between 8.5 and 9.0 mm mean diameter. It therefore seems likely that this rather low hypoxic fraction observed in small intramuscular tumours is attributable to the size of the tumours used, rather than to differences in the tumour as used in different laboratories, although the latter cannot be ruled out. The main conclusion from the radiosensitivity studies was therefore that the increase in hypoxic fraction was very similar, irrespective of the type of tumours or its initial hypoxic fraction.

Prolonged hypoxia and/or reduced pH thermosensitise cells *in vitro* (Hahn, 1974; Gerweck et al., 1979; Overgaard & Bichel, 1977; Overgaard & Neilsen, 1980); however since hypoxia typically results in decreased pH, it can be difficult to determine the relative contributions of these interdependent factors to the ultimate toxic effect, particularly *in vivo*. Acute hypoxia induced by inhibition of blood flow has also been shown to potentiate thermal damage in two mouse tumours (Horsman et al., 1989, Honess et al., 1991*a*). These observations form the rationale for testing BW12C as a potentiator of thermal killing *in vivo*. We have previously demonstrated that the drug does not thermosensitise cells *in vitro* (Honess, unpublished observations) and have also shown that BW12C does not thermosensitise the RIF-1 tumour in the leg (Honess et al., 1989). However in this tumour the hypoxic fraction is only increased to 1.5% by BW12C (Table I). Clearly a tumour with a higher hypoxic fraction would be better system in which to test the hypothesis that BW12C may thermosensitise tumours, and the KHT tumour was selected on the basis of published data on its hypoxic fraction (see above). Leg tumour size was very carefully controlled within the same limits for both the thermosensitivity and radiosensitivity experiments, and these limits were determined by the requirements of the hyperthermia system in order to ensure uniformity of temperature. The radiosensitivity data showed that the hypoxic fraction of the KHT leg tumours was increased to around 4% by BW12C, which was significantly higher than the hypoxic fraction of 1.5% for BW12C-treated RIF-1 in the leg, but far from full radiobiological hypoxia. The data presented in the Results section show that BW12C did not increase the thermal damage produced by heat at temperatures from 43°C to 44°C. The small but measurable growth delay caused by BW12C alone is at first sight in contrast to the absence of cell killing by this dose of drug seen in the cell survival studies. However, the drug did cause marked reduction in blood flow (Figure 2) and hence deprivation of nutrition for a period sufficient to check the growth of this rapidly growing tumour. In studies with hydralazine, using this tumour system in this site, we have previously shown that growth delay is closely related to duration of inhibition of blood flow in addition to its dependence on cell killing (Honess et al., 1991*a*). We conclude from the present data that the degree of hypoxia induced by BW12C was inadequate to alter the thermal response of the tumour. We were unable to investigate the effect of heat on the tumour in the flank as the hyperthermia system is designed only for the treatment of leg tumours.

The comparative data on the effects of BW12C on relative perfusion of RIF-1 tumours grown in the leg and flank (Figure 2) confirm the preliminary report (Honess et al., 1989) that perfusion is reduced. The data strongly suggest that the reduction in perfusion contributes to the increase in hypoxic fraction demonstrated to Figure 1 and Table I. The time-course of the perfusion effects is such that perfusion approaches its nadir during irradiation, which starts 30 min after drug administration and continues for e.g. approximately 30 min for 20 Gy. We have previously shown that maximum radioprotection of the RIF-1 tumour occurs when radiation is started around 30 min after BW12C administration, whereas the maximum haemoglobin modification is observed 5 min after giving BW12C and this modification decays with a half-life of about 1.25 h (Honess et al., 1991*b*). The development of radioprotection therefore appears to correlate rather better with the time-course of the perfusion changes than with the haemoglobin modification. The data are consistent with radioprotective hypoxia initially (15 min after giving the drug) being primarily due to altered blood chemistry, then as the haemoglobin alteration decays (1 h after the drug) the effect of blood flow reduction becomes relatively more important in maintaining hypoxia. By 2 h after the drug, when blood flow has normalised and only 25% of the blood is modified, all radioprotection is lost.

The present data show that the reduction in perfusion in the leg tumours appears to be secondary to a reduction in perfusion of the muscle, the tissue 'feeding' the tumour, while for the intradermal flank tumours the reduction is not dependent on the perfusion of the skin. The reduction in muscle perfusion was not significant in the preliminary experiments (Honess et al., 1989), where absolute values for per cent injected activity per gram were pooled rather than per cent of mean control values and where there was greater variation in the reproducibility of control values than in the present series of experiments; this probably obscured the changes in muscle. The reason for the difference in response between intramuscular and intradermal tumours and their respective upstream tissues is not clear. However, the proportional changes in perfusion are broadly similar in both sites, with a larger reduction in the flank tumours. This is consistent with the larger increase in hypoxic fraction measured for flank than for leg tumours (Table I) although there is no evidence for a significant difference between the increases in flank and leg.

The time course for change in relative perfusion after BW12C in the remaining normal tissues measured was somewhat similar in that a peak at either 30 min or 1 h was followed by a drop at 2 h. However, in kidney, spleen and lung this peak represented an increase in relative perfusion followed by return to normal values, whereas in liver the peak did not form a significant increase, but the subsequent fall constituted a significant decrease from control values (Figure 2). The rises observed may either reflect actual increases in absolute blood flow, or possibly may indicate a decrease in cardiac output at around 1 h. Relative tissue perfusion is a measure of absolute blood flow as a proportion of cardiac output. Hence RTP must inevitably rise when cardiac output falls but absolute blood flow remains unchanged. Similarly a reduction in cardiac output by the same proportion as a reduction in blood flow will cause no change in RTP, but a reduction in blood flow by a greater proportion than a reduction in cardiac output will be measured as a decrease in RTP. The similarity of pattern of RTP changes in kidney, spleen, lung and liver suggested that the changes in RTP might be due to a drop in cardiac output with a nadir at 1 h after BW12C and recovery commencing by 2 h. It is not currently feasible to measure cardiac output in unanaesthetised mice, so a direct measurement was not possible. Functional assays of the effects of BW12C on these four organs were considered, and we chose to examine the effects of BW12C on renal clearance. The data presented in Table II shows that 30 min after BW12C, the time at which RTP was maximal, there was no effect on EDTA or $^{125}$I-iodohippurate clearance, while immediately after giving the drug there was a very small decrease in EDTA clearance rate, by a factor ($\pm$ 2 s.e.) of $1.26 \pm 0.27$. EDTA clearance is used as an assay for glomerular filtration rate and iodohippurate clearance as an assay for effective renal plasma flow (Sweny *et al.*, 1989). These experiments showed that kidney function was hardly altered by BW12C, and a possible inference is that the RTP measurements may not indicate an increase in absolute kidney flow but a decrease in cardiac output. The RTP data are compatible with this hypothesis, but there is no clear evidence to support it. Nonetheless, if it is true, since any change in cardiac output is a constant in the determination of RTP in all tissues measured, the absolute reductions in flow in tissues where RTP dropped must have been larger than the measured reductions in RTP i.e. the reductions in tumour RTP would be underestimates of the changes in absolute tumour blood flow.

In summary, BW12C induces a comparable increase in hypoxic fraction in both the RIF-1 and KHT tumours, whether these tumours are grown intramuscularly in the leg (with a low hypoxic fraction) or intradermally or subcutaneously in the flank (with a hypoxic fraction 15 to 17-fold higher). The blood flow studies indicate that a reduction in tumour perfusion is likely to be an important factor in this increase in hypoxic fraction. It is not clear from these studies how much of the radioprotection is attributable to the reduction in perfusion and how much is due to changes in oxygen release by alteration of blood chemistry by BW12C.

We are very grateful to Ms Angela Prime for expert technical assistance.

### References

ADAMS, G.E., BARNES, D.W.H., DU BOULAY, C. & 10 others (1986). Induction of hypoxia in normal and malignant tissues by changing the oxygen affinity of haemoglobin – implications for therapy. *Int. J. Radiat. Oncol. Biol. Phys.*, **12**, 1299.

ADAMS, G.E., STRATFORD, I.J., NETHERSELL, A.B.W. & WHITE, R.D. (1989). Induction of severe tumour hypoxia by modifiers of the oxygen affinity of haemoglobin. *Int. J. Radiat. Oncol. Biol. Phys.*, **16**, 1179.

BEDDELL, C.R., GOODFORD, P.J., KNEEN, G., WHITE, R.D., WILKINSON, S. & WOOTTON, R. (1984). Substituted benzaldehydes designed to increase the oxygen affinity of human haemoglobin and inhibit the sickling of sickle erythrocytes. *Br. J. Pharmacol.*, **82**, 397.

BROWN, J.M., TWENTYMAN, P.R. & ZAMVIL, S.S. (1980). Response of the RIF-1 tumour *in vitro* and in C3H/Km mice to X-radiation (cell survival, regrowth delay and tumour control), chemotherapeutic agents and activated macrophages. *J. Natl Cancer Inst.*, **64**, 605.

COLE, S. & ROBBINS, L. (1989). Manipulation of oxygenation in a human tumour xenograft with BW12C or hydralazine: effects on responses to radiation and to the bioreductive cytotoxicity of misonidazole or RSU-1069. *Radiother. Oncol.*, **16**, 235.

FITZHARRIS, P., McLEAN, A.E.M., SPARKS, R.G., WEATHERLEY, B.C., WHITE, R.D. & WOOTTON, R. (1985). The effects in volunteers of BW12C, a compound designed to left-shift the blood-oxygen saturation curve. *Br. J. Pharmacol.*, **19**, 471.

GERWECK, L.E., NYGAARD, T.G. & BURLETT, M. (1979). Response of cells to hyperthermia under acute and chronic hypoxic conditions. *Cancer Res.*, **39**, 966.

HAHN, G.M. (1974). Metabolic aspects of the role of hyperthermia in mammalian cell inactivation and their possible relevance to cancer treatment. *Cancer Res.*, **34**, 3117.

HILL, R.P. (1980). An appraisal of *in vivo* assays of excised tumours. *Br. J. Cancer*, **41**, Suppl IV, 230.

HONESS, D.J. & BLEEHEN, N.M. (1982). Sensitivity of normal mouse marrow and RIF-1 tumour to hyperthermia combined with cyclophosphamide or BCNU: a lack of therapeutic gain. *Br. J. Cancer*, **46**, 236.

HONESS, D.J. & BLEEHEN, N.M. (1991). Comparative effects of hydralazine on KHT tumour, kidney and liver perfusion and on renal function in mice. *Int. J. Radiat. Oncol. Biol. Phys.* (in press).

HONESS, D.J., HU, D.E. & BLEEHEN, N.M. (1991*a*). A study of the mechanism of hydralazine enhancement of thermal damage in the KHT tumour. *Int. J. Hyperthermia*, **7**, 667.

HONESS, D.J., NETHERSELL, A.B.W. & BLEEHEN, N.M. (1991*b*). *In vitro* and *in vivo* studies on BW12C: toxicity, haemoglobin modification and effects on the radiosensitivity of normal marrow and RIF-1 tumours in mice. *Int. J. Radiat. Biol.* (in press).

HONESS, D.J., WHITE, R.D., NETHERSELL, A.B.W. & BLEEHEN, N.M. (1989). Effects of the manipulation of oxyhaemoglobin status by BW12C on tumor thermosensitivity and on blood flow in tumor and normal tissues in mice. *Int. J. Radiat. Oncol. Biol. Phys.*, **16**, 1187.

HORSMAN, M.R., CHRISTENSEN, K.L. & OVERGAARD, J. (1989). Hydralazine-induced enhancement of hyperthermic damage in a C3H mammary carcinoma *in vivo*. *Int. J. Hyperthermia*, **5**, 123.

KALLMAN, R.F., SILINI, G. & VAN PUTTEN, L.M. (1967). Factors influencing the quantitative estimation of the *in vivo* survival of cells from solid tumours. *J. Natl Cancer Inst.*, **39**, 359.

KEIDAN, A.J., WHITE, R.D., HUEHNS, E.R., FRANKLIN, I.M., JOY, M. & STUART, J. (1986). Effect of BW12C on oxygen affinity of haemoglobin in sickle-cell disease. *Lancet*, **i**, 831.

KENNEDY, K.A. (1987). Hypoxic cells as specific targets for chemotherapy. *Anticancer Drug Design*, **2**, 181.

MOULDER, J.E. & ROCKWELL, S. (1984). Hypoxic fractions of solid tumours: experimental techniques, methods of analysis, and a survey of existing data. *Int. J. Radiat. Oncol. Biol. Phys.*, **10**, 695.

OVERGAARD, J. & BICHEL, P. (1977). The influence of hypoxia and acidity on the hyperthermic response of malignant cells *in vitro*. *Radiology*, **123**, 511.

OVERGAARD, J. & NEILSEN, O.S. (1980). The role of tissue environment factors on the kinetics and morphology of tumour cells exposed to hyperthermia. *Ann. NY Acad. Sci.*, **335**, 254.

RAMSAY, J.R.S., BLEEHEN, N.M., FALK, S.J. & 5 others (1991). Phase 1 study of BW12C in combination with mitomycin C in patients with gastrointestinal cancer. *Int. J. Radiat. Oncol. Biol. Phys.* (in press).

SAPIRSTEIN, L.A. (1959). Regional blood flow by fractional distribution of indicators. *Am. J. Physiol.*, **193**, 161.

SARTORELLI, A.C. (1988). Therapeutic attack of hypoxic cells of solid tumours: presidential address. *Cancer Res.*, **48**, 775.

SIEMANN, D.W., ALLIET, K.L. & MACLER, L.M. (1989). Manipulations in the oxygen transport capacity of blood as a means of sensitising tumors to radiation therapy. *Int. J. Radiat. Oncol. Biol. Phys.*, **16**, 1169.

SIEMANN, D.W., HILL, R.P. & BUSH, R.S. (1978). Smoking: the influence of carboxyhaemoglobin (HbCO) on tumor oxygenation and response to radiation. *Int. J. Radiat. Oncol. Biol. Phys.*, **4**, 657.

SIEMANN, D.W. & KENG, P.C. (1987). Characterisation of radiation resistant hypoxic cell subpopulations in KHT sarcomas. (1) Centrifugal elutriation. *Br. J. Cancer*, **55**, 33.

SIEMANN, D.W. & MACLER, L.M. (1986). Tumor radiosensitisation through reductions in hemoglobin affinity. *Int. J. Radiat. Oncol. Biol. Phys.*, **12**, 1295.

STRATFORD, I.J., O'NEILL, P., SHELDON, P.W., SILVER, A.R.J., WALLING, J.M. & ADAMS, G.E. (1986). RSU-1069, a nitroimidazole containing an aziridine group: bioreduction greatly increases cytotoxicity under hypoxic conditions. *Biochem. Pharmacol.*, **36**, 105.

SWENY, P., FARRINGTON, K. & MOORHEAD, J.F. (1989). Renal blood supply and its regulation. In *The Kidney and its Disorders*, p. 16. Blackwell Scientific Publications: Oxford.

TWENTYMAN, P.R., BROWN, J.M., GRAY, J.W., FRANKO, A.J., SCOLES, M.A. & KALLMAN, R.F. (1980). A new mouse model tumour system (RIF-1) for comparison of end-point studies. *J. Natl Cancer Inst.*, **64**, 595.

VAN DEN AARDWEG, G.J.M.J., HOPEWELL, J.W., ADAMS, G.E. & 4 others (1991). Protection of the pig epidermis against radiation-induced damage by infusion of BW12C. *Int. J. Radiat. Biol.*, **59**, 1039.

WALTON, M.I., CATTERMOLE, D. & BLEEHEN, N.M. (1989). A microcomputer-controlled, local hyperthermia system for uniform tumour heating in unanaesthetised mice. *Int. J. Hyperthermia*, **5**, 53.

ZEMAN, E.M., BROWN, J.M., LEMMON, M.J., HIRST, V.K. & LEE, W.W. (1986). SR4233: a new bioreductive agent with a high selective toxicity for hypoxic mammalian cells. *Int. J. Radiat. Oncol. Biol. Phys.*, **12**, 1239.